# Comparison of British Thyroid Association, American College of Radiology TIRADS and Artificial Intelligence TIRADS with histological correlation: diagnostic performance for predicting thyroid malignancy and unnecessary fine needle aspiration rate

**DOI:** 10.1259/bjr.20201444

**Published:** 2021-06-09

**Authors:** Linda Watkins, Greg O'Neill, David Young, Claire McArthur

**Affiliations:** 1 Department of Radiology, Glasgow Royal Infirmary, NHS GG&C, Glasgow, UK; 2 Departmentof Mathematics and Statistics, Strathclyde University, Glasgow, UK; 3 University of Glasgow, Glasgow, UK

## Abstract

**Objectives:**

To compare diagnostic performance of British Thyroid Association (BTA), American College of Radiology Thyroid Imaging Reporting and Data System (ACR-TIRADS) and Artificial Intelligence TIRADS (AI-TIRADS) for thyroid nodule malignancy. To determine comparative unnecessary fine needle aspiration (FNA) rates.

**Methods:**

218 thyroid nodules with definitive histology obtained during 2017 were included. Ultrasound images were reviewed retrospectively in consensus by two subspecialist radiologists, blinded to histopathology, and nodules assigned a BTA, ACR-TIRADS and AI-TIRADS grade. Nodule laterality and size were recorded to allow accurate histopathological correlation and determine which nodules met criteria for FNA.

**Results:**

77 (35.3%) nodules were malignant. Deeming ultrasound Grade 4–5 as test-positive and 1–2 as test-negative, sensitivity and specificity for BTA was 98.28 and 42.55%, for ACR-TIRADS: 95.24 and 40.57% and for AI-TIRADS: 93.44 and 45.71%. FNA was indicated in 101 (71.6%), 67 (47.5%) and 65 (46.1%) benign nodules utilising BTA, ACR-TIRADS and AI-TIRADS respectively. The unnecessary FNA rate was significantly higher with BTA (46.3%) compared to ACR-TIRADS (30.7%) and AI-TIRADS (29.8%) *p* < 0.001.

**Conclusion:**

BTA, ACR-TIRADS and AI-TIRADS had similar diagnostic performance for predicting thyroid nodule malignancy with sensitivity >93% for all systems when considering ultrasound Grade 4–5 as malignant and Grade 1–2 as benign. ACR-TIRADS and AI-TIRADS both had a significantly lower rate of recommended FNA in benign nodules compared to BTA.

**Advances in knowledge:**

BTA, ACR-TIRADS and AI-TIRADS have comparable diagnostic performance with high sensitivity but relatively low specificity for predicting thyroid nodule malignancy in this cohort using histology as gold-standard. Using Grade 1–2 as benign and 4–5 as malignant there were more false negatives with TIRADS but this improved when taking other features into account while BTA had a significantly higher rate of unnecessary FNA.

## Introduction

Thyroid nodules occur commonly with incidental nodules found in up to 67% of the population examined with high-resolution ultrasound.^1^ Between 5 and 15% of nodules turn out to be malignant depending on age, sex, radiation exposure, family history and other factors.^2^ Following introduction of neck ultrasound and image-guided FNA, there has been a worldwide increase in the incidence of thyroid cancer over the past four decades. Mortality rates however have remained stable. The increase is chiefly accounted for by small papillary cancers. These small cancers, especially those <1 cm, are very often indolent, rarely becoming symptomatic or fatal.^3,4^ Thus, finding a thyroid nodule frequently poses a diagnostic dilemma. The aim of ultrasound is to assist in identification of biologically relevant cancers, whilst avoiding needless investigation and overdiagnosis of clinically non-significant nodules.

A number of sonographic parameters have been shown to reliably correlate with thyroid malignancy, including nodule solidity, hypoechogenicity, taller than wide dimensions in the transverse plane, lobulation or spiculation and microcalcifications. Fine needle aspiration (FNA) is frequently required next to further assess whether a nodule is malignant, benign or requires surgery for definitive diagnosis. A variety of ultrasound grading systems have been developed which stratify the risk of malignancy and identify nodules requiring FNA.

In July 2014, the British Thyroid Association (BTA) published guidelines on thyroid cancer management, in which they recommended a U1 to U5 ultrasound grading to assess risk of malignancy in thyroid nodules.^5,6^ Nodule characteristics for U1-5 categories are shown In Table 1. FNA is recommended for nodules graded U3 or above.^5^ A recent study demonstrated the reliability of the BTA classification although it remained undefined as to the appropriate interval and duration of follow-up for indeterminate nodules without high-risk cytology or clinical features.^7^ An earlier validation study found it to be a robust method but only a small proportion of studied nodules had cytological or histological correlation.^8^

**Table 1.** Description of BTA U1-5 category appearances^5,6^

U1	U2	U3	U4	U5
Normal	Microcystic/spongiform	Homogeneous, hyperechoic (markedly), solid, halo (follicular lesion)	Solid, hypoechoic	Solid, hypoechoic, lobulated/irregular outline, microcalcification (?papillary carcinoma)
	Halo, iso/mildly hyperechoic	?Hypoechoic, equivocal echogenic foci, cystic change	Solid, very hypoechoic	Solid, hypo-echoic, lobulated/irregular outline, globular calcification (?medullary carcinoma)
	Peripheral eggshell calcification	Mixed/central vascularity	Disrupted peripheral calcification, hypoechoic	Intra nodular vascularity
	Cystic change ± ring down sign (colloid)		Lobulated outline	Shape (taller >wide) (AP >TR)
	Peripheral vascularity			Characteristic associated lymphadenopathy

Following introduction by Horvath^9^ and Park^10^ with subsequent modification by Kwak,^11^ the American College of Radiology (ACR) proposed a Thyroid Imaging Reporting and Data System (TIRADS) in 2017 using five ultrasound categories.^12^ These comprise nodule composition, echogenicity, shape, margin and echogenic foci where each feature is assigned between 0 and 3 points depending on the findings, with total points determining malignancy risk level from TR1 (benign) to TR5 (highly suspicious). In combination with maximum nodule diameter, the TR level indicates whether to recommend FNA, a follow-up ultrasound strategy or no further action (Table 2). It is designed to identify the majority of clinically significant malignancies while reducing the number of biopsies performed on benign nodules.

**Table 2.** Thresholds for FNA or follow-up by category with ACR-TIRADS and AI-TIRADS

TIRADS grad	FNA indicated	Follow-up indicated
TR 1	no	No
TR 2	no	No
TR 3	≥2.5 cm	≥1.5 cm 1,3 and 5 years
TR 4	≥1.5 cm	≥1 cm 1,2,3 and 5 years
TR 5	≥1 cm	≥0.5 cm annual for up to 5 years

ACR-TIRADS, American College of Radiology Thyroid Imaging Reporting and Data System; AI-TIRADS, Artificial Intelligence TIRADS; FNA, fine needle aspiration.

Artificial intelligence (AI), or deep learning, has a rapidly developing role in multiple facets of imaging. AI-TIRADS, recently proposed by Wildman-Tobriner et al^13^, was developed by using a training set of thyroid nodules with known outcomes using an AI algorithm in an effort to refine the ACR-TIRADS system. This modified scoring system grants a lower score than ACR-TIRADS for any non-solid nodules, those with hyper- or isoechoic solid components, taller-than-wide shape or macrocalcifications but a higher score for solid nodules. Thereafter, thresholds for FNA or follow-up are based on score and size as per ACR-TIRADS.

A systematic review published earlier this year^14^ regarding sites using the UK Royal College of Pathology reporting system found a non-diagnostic, *i.e*. Thy1, cytology rate ranging from 3.0 to 43.3%. With a national Thy1 rate of 20–29%^15^ repeat attempts at FNA are often required. An ideal ultrasound classification system would have good accuracy with a low rate of FNA indicated in ultimately benign nodules.

The study aims were to comparatively evaluate BTA, ACR-TIRADS and AI-TIRADS in assessing malignancy risk in a cohort of thyroid nodules with definitive histology and to compare the unnecessary FNA rate with each method.

## Methods and materials

### Ethical considerations

Research ethics committee advice was sought using the online tool from the NHS health research authority and Medical Research council website^16^ and was not required.

### Subjects

Of 237 patients in a large health board who had undergone pre-operative thyroid ultrasound with eutopic thyroid histology results available between January 1, 2017 and December 31, 2017, 212 patients were included. 25 patients were excluded due to ultrasound demonstrating diffuse thyroid disease such as thyroiditis or diffuse multinodular goitre rather than a discrete nodule (14) or if it was not considered possible to reliably correlate imaging and histopathology, due to, *e.g.* suboptimal image quality (11). A total of 218 nodules were analysed, with the majority of patients having 1 nodule assessed while 6 patients had 2 nodules evaluated.

Based on histopathology and ultrasound reports, laterality (left, right, both or isthmus), nodule size and pathology outcome of the target nodules were recorded by Author 1 (LW) who has 2 years of general radiology experience.

Each patient was assigned a unique anonymous code on a Picture Archiving Communication System (PACS) (Carestream Health Inc. Rochester, NY) custom worklist. The anonymised static ultrasound images were examined retrospectively by two head and neck subspecialist Consultant Radiologists (authors 2 and 4, GO and CM) with 15 and 7 years’ experience. If there was more than one ultrasound attendance, the latest prior to nodule FNA sampling was chosen. If no FNA, the most recent ultrasound prior to surgery was used. The original ultrasound examinations were performed on a range of ultrasound machines across several institutions within the hospital trust by 1 of 12 consultant radiologists or 1 of 2 sonographers, all with experience of thyroid ultrasound. The study observers were blinded to the histopathology results but were made aware of the relevant nodule laterality.

### Nodule assessment

Using the BTA and ACR-TIRADS guidelines, the index nodule(s) were scored in consensus. The maximum diameter of these nodules was measured using calipers on the ultrasound images to aid cross-referencing with histology results. An assessment of nodule composition was made, whether completely or almost completely solid, spongiform, mixed composition, or completely or almost entirely cystic. Any solid element was described as hyper-, iso- or hypoechoic in relation to thyroid parenchyma or “markedly hypoechoic” in relation to strap muscle. Note was made if the nodule was taller than wide in the transverse plane and whether there was irregularity, lobulation, spiculation or frank extrathyroidal extension. The presence of comet tail artefacts suggesting colloid, punctate echogenic foci more suggestive of microcalcifications, equivocal echogenic foci, rim calcification or macrocalcification was recorded. Prominent intranodular vascularity was noted if evident. Presence of pathological looking cervical nodes was also detailed. AI-TIRADS grading was obtained by modifying the scores for each category from the ACR-TIRADS proforma. In addition, note was made if there was difficulty in categorising the nodule with any of the classifications.

### Data and statistical analysis

The nodule grading for each system was compared with histopathology results. Histopathology was considered the gold-standard and for the purposes of analysis, nodules were classified in binary fashion as benign or malignant. Papillary microcarcinoma (PMC) was only labelled a malignant case if it definitely corresponded with the subject nodule. More often PMCs were found incidentally following surgery for a larger subject nodule. Non-invasive follicular thyroid neoplasms with papillary-like nuclear features (NIFTPs) were deemed benign.

Malignancy rate was determined for each ultrasound category and expressed as a percentage of the nodules in that category. Diagnostic performance, *i.e*. sensitivity, specificity, positive predictive value (PPV), negative predictive value (NPV) and accuracy of the three classifications were determined along with 95% confidence intervals. For this, scores of BTA U2 or ACR-TIRADS and AI-TIRADS TR1 and TR2 were regarded as test negative and scores of BTA U4 and U5 and ACR and AI-TIRADS TR4 and TR5 were grouped as test positive. The nodules scored as U3 or TR3 were designated as indeterminate for the purposes of assigning true and false positives and negatives, and therefore were not included in this part of the analysis. Post-hoc recommendations for further management, including FNA, for index nodules were assigned on the basis of ultrasound score and, where applicable, nodule diameter. If FNA would not have been recommended by applying the TIRADS guidelines, then it was recorded whether ultrasound follow-up would have been indicated. Proportion of FNAs recommended in benign and malignant nodules was recorded as a percentage. The term “unnecessary FNA”, for brevity, refers to FNA being recommended in an ultimately benign nodule. The rate was calculated as the number of histologically benign nodules with FNA recommendation divided by the total number of nodules.

All statistical analyses were done using MedCalc (v. 19)^17^ and Minitab (v. 18)^18^ with a 5% significance level.

## Results

Thyroid histology was obtained in 212 adult patients during the study period following unilateral hemithyroidectomy (152), total thyroidectomy (37), core biopsy alone (8), completion thyroidectomy (5), subtotal thyroidectomy (4), isthmusectomy (3) and nodulectomy (3).

Nodule diameter as measured on ultrasound was mean 19 mm ± 19.1 (range 5–100 mm) with 16 nodules (7%) <1 cm. 161 patients were female (76%) and patient age was mean 58.5 years ± 29 (range 17–86 years).

Of the 218 graded nodules, 141 were confirmed benign (64.7%) and 77 confirmed malignant (35.3%). Histological findings are outlined inTable 3 . In 23 benign cases and 7 malignant cases, additional incidental PMC was also found.

**Table 3.** Histological Type of All Nodules

Histological types	Number
*Malignant (77*)	
Papillary carcinoma	48
Follicular carcinoma	9
Papillary cicrocarcinoma	5
Medullary carcinoma	4
Poorly differentiated carcinoma	3
Squamous cell carcinoma (primary)	3
Anaplastic carcinoma	2
Hurthle cell carcinoma	1
Lymphoma	1
Metastasis to thyroid	1
*Benign (141*)	
Follicular adenoma	71
Hyperplastic nodules/Multinodular goitre	43
NIFTP	11
Adenomatoid nodules	7
Degenerate benign nodules	3
Nodular Hashimoto’s	2
Intrathyroidal parathyroid adenoma	2
Foregut duplication cyst	1
Organising haematoma	1

**Figure 1.**
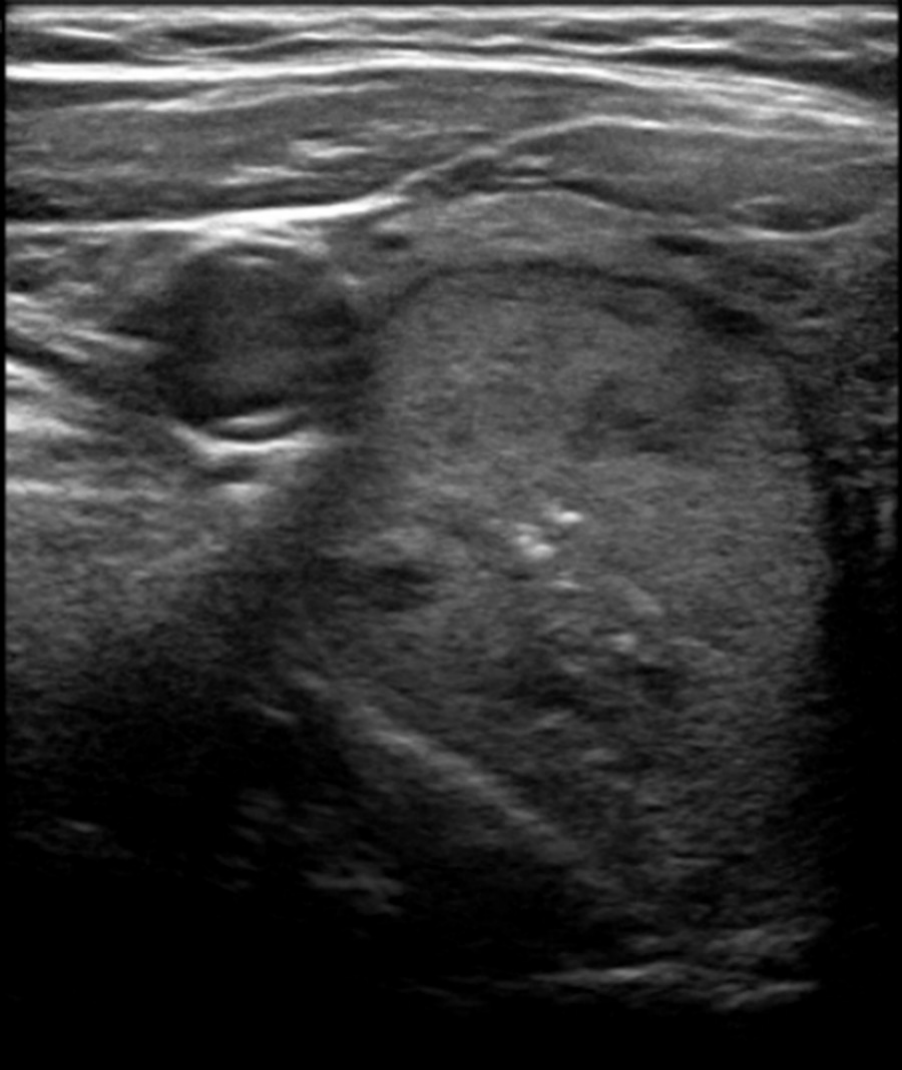
Axial ultrasound image showing a predominantly solid, isoechoic nodule in the right lobe with central punctate echogenic foci which was more difficult to classify using BTA. BTA, British Thyroid Association

In 12 (5.5%) cases, the index nodule was noted to be difficult to classify or to reach a consensus agreement using BTA scoring. For example, nodules that were solid or mixed composition and isoechoic but with either punctate echogenic foci or lobulated margin (Figure 1), or nodules that had overall benign characteristics but had prominent intranodular vascularity. In such cases, the best fit was used considering what grading would most likely be applied in actual clinical practice. No nodules were considered difficult to grade using ACR or AI TIRADS.

Table 4 shows the consensus grading with each classification for benign and malignant nodules. Diagnostic performance for each classification is displayed in Table 5. Applying BTA, ACR-TIRADS and AI-TIRADS respectively, 71.6, 47.5 and 46.1% of benign nodules and 98.7, 79.2 and 76.6% of malignant nodules would have met FNA criteria (Table 6). This resulted in a significantly (χ^2^ *p* < 0.001) higher unnecessary FNA rate of 46.3% when applying BTA compared to both ACR (30.7%) and AI-TIRADS (29.8%).

**Table 4.** Malignancy rate with each ultrasound classification category

Classification and score	Benign nodules *n* = 141	Malignant nodules *n* = 77	Malignancy rate	Recommended malignancy rate^19^
BTA				
U2	40	1	2.4%	
U3	47	19	28.8%	
U4	43	29	40.3%	
U5	11	28	71.8%	
ACR-TIRADS				
TR1	14	0	0%	0.3%
TR2	29	3	9.4%	1.5 %
TR3	35	14	28.6%	4.8%
TR4	43	25	36.8%	9.1%
TR5	20	35	63.6%	35%
AI-TIRADS				
TR1	44	3	6.4%	
TR2	4	1	20.0%	
TR3	36	16	30.8%	
TR4	31	19	38.0%	
TR5	26	38	59.4%	

BTA, British Thyroid Association.

ACR-TIRADS, American College of Radiology Thyroid Imaging Reporting and Data System; AI-TIRADS, Artificial Intelligence TIRADSxMark as

**Table 5.** Diagnostic Performance Indices for each category excluding BTA U3 and ACR-TIRADS/AI-TIRADS TR3 nodules

	BTA (*n* = 152)	ACR-TIRADS (*n* = 169)	AI-TIRADS (*n* = 166)
Sensitivity (%)	98.28 (90.76, 99.96)	95.24 (86.71, 99.01)	93.44 (84.05, 98.18)
Specificity (%)	42.55 (32.41, 53.18)	40.57 (31.13, 50.54)	45.71 (35.96, 55.72)
PPV (%)	51.35 (46.92, 55.76)	48.78 (44.63, 52.94)	50.00 (45.32, 54.68)
NPV (%)	97.56 (84.96, 99.65)	93.48 (82.27, 97.79)	92.31 (55.43, 70.59)
Accuracy (%)	63.82 (55.64, 71.44)	60.95 (53.16, 68.35)	63.25 (55.43, 70.59)

ACR-TIRADS, American College of Radiology Thyroid Imaging Reporting and Data System; AI-TIRADS, Artificial Intelligence TIRADS; BTA, British Thyroid Association; NPV, negative predictive value; PPV, positive predictive value.

**Table 6.** Nodules in benign and malignant group where FNA was recommended and unnecessary FNA rate

Classification	FNA in benign nodules *n* = 141	FNA in malignant nodules *n* = 77	Unnecessary FNA rate (Benign nodule FNA/total number of nodules)
BTA	101 (71.6%)	76 (98.7%)	46.3%
ACR TIRADS	67 (47.5%)	61 (79.2%)	30.7%
AI-TIRADS	65 (46.1%)	59 (76.6%)	29.8%

ACR-TIRADS, American College of Radiology Thyroid Imaging Reporting and Data System; AI-TIRADS, Artificial Intelligence TIRADS; BTA, British Thyroid Association; FNA, fine needle aspiration.

**Figure 2.**
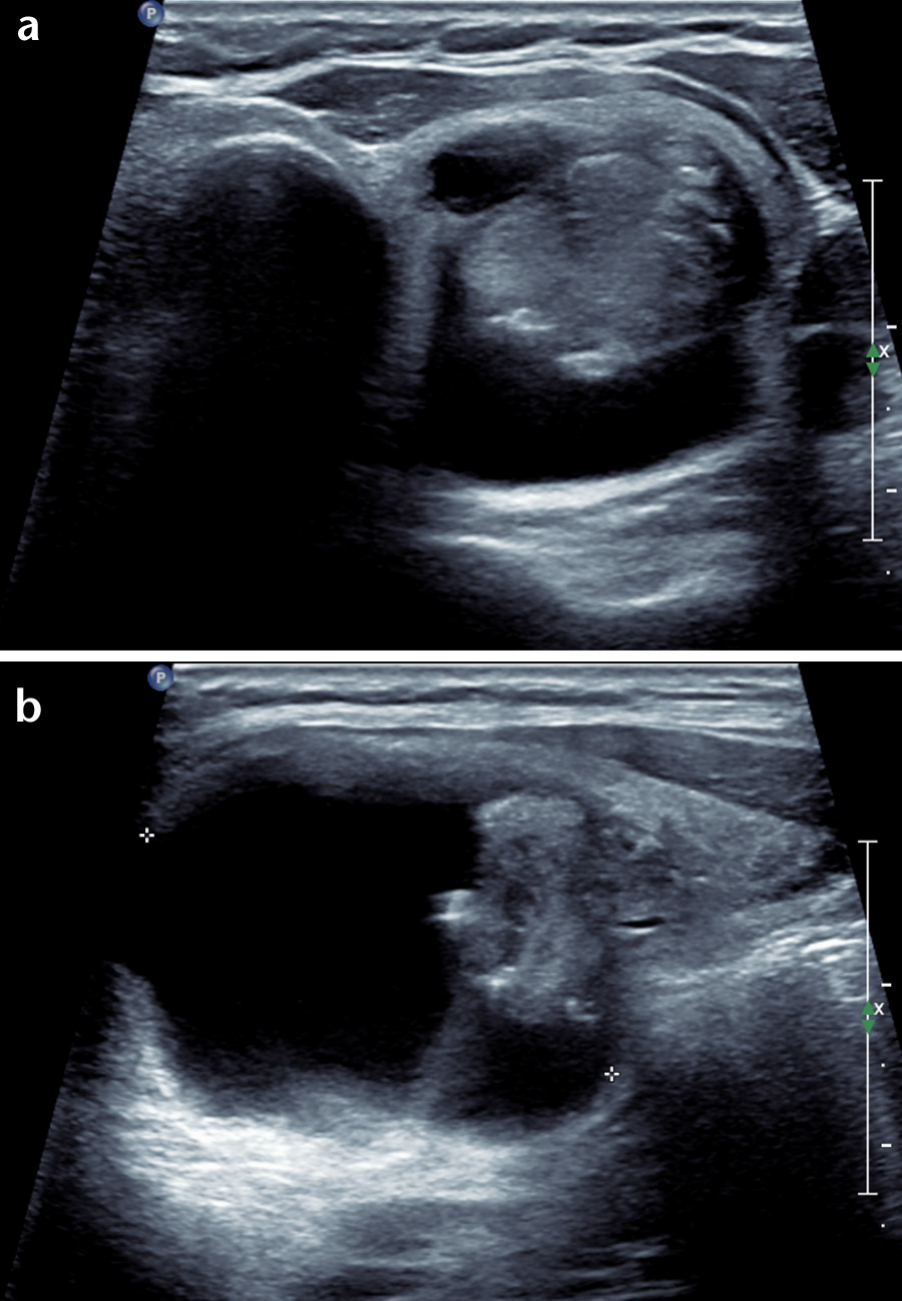
Axial (**a**) and longitudinal (**b**) ultrasound of a papillary carcinoma in the left lobe: a well-defined mixed composition nodule with isoechoic solid component however the solid focus is eccentrically positioned forming a mural nodule that has an acute angle with the cyst wall.

The majority of malignant nodules below FNA threshold had follow-up ultrasound recommended at an initial 1 year interval with both TIRADS systems. Malignant nodules that would have required no FNA or follow-up comprised 6/77 (7.8%) using ACR-TIRADS, 7/77 (9.1%) using AI-TIRADS and 1/77 (1.3%) using BTA. Of those not meeting criteria for FNA or follow-up with ACR-TIRADS, three were false-negatives, categorised TR2, simultaneously TR1 with AI-TIRADS and U3 with BTA. These were mixed composition with isoechoic solid elements although two of these had a mural nodule, the margins of which formed an acute angle in relation to the nodule wall. An example is shown in Figure 2. Histology of these three cases was oncocytic variant papillary carcinoma (one), mixed classical, follicular and columnar variant papillary carcinoma (one) and squamous cell carcinoma (one). A further malignant nodule was graded simultaneously ACR and AI-TIRADS TR3 and BTA U3 but below size limit for FNA with TIRADS, a follicular variant papillary carcinoma while two further cases were graded TR4 with both TIRADS methods and did not meet criteria for FNA or follow-up at 8 and 9 mm diameter. A further malignant nodule graded TR2 with AI-TIRADS was simultaneously graded TR4, for FNA with ACR-TIRADS and U3 with BTA, a papillary carcinoma. With BTA, there was one false negative 11 mm nodule graded U2 but considered TR4 under both TIRADS schemes and thus would have had proposed ultrasound follow-up. This was a papillary carcinoma.

## Discussion

This retrospective study compares performance of three ultrasound classification systems for prediction of malignancy and guiding further investigation in thyroid nodules. These three systems have not previously been directly compared in the available literature.

As expected, increasing ultrasound grade resulted in increasing likelihood of malignancy. Overall malignancy rate in our study (35.3%) was higher than in some other studies where malignancy rates ranged from 4.9 to 22.7%^8,20–22^ Malignancy proportion within each category was also notably higher than that previously published with ACR-TIRADS.^19^ Our higher rate is closer to Chng et al. (31%)^23^ who used only surgically resected cases, and Ha et al^24^ (37.2%). This is almost certainly explained by the fact that we used surgical excision or, in a small number core biopsy, histology as the gold-standard. Patients are far more likely to undergo resection if there are non-diagnostic, indeterminate or suspicious findings on cytology, higher nodule grade on ultrasound or concerning patient clinical history and examination. The majority of other comparative and validation studies correlate ultrasound scoring with a combination of cytology, histology and ultrasound follow-up. As we were careful to avoid classifying incidental PMCs automatically as malignant cases, rather we based categorisation on the histology of the index nodule, this should not have affected the malignancy rate.

Sensitivity and NPV were generally high across all classifications with accuracy comparable between each method. Probably the closest comparison of systems to ours was published by Chng et al^23^ who compared American Thyroid Association (ATA), BTA and the previously proposed Kwak TIRADS system where each had sensitivity >90% and good NPV. Kim et al^25^ found sensitivity >90% for TR 4/5 nodules with ACR, Korean and European TIRADS. AI-TIRADS was previously and again more recently found to have equally good sensitivity as ACR-TIRADS but improved specificity.^13,22^

There was some difficulty in classifying all nodules in the current study using BTA classification. Several studies have noted that a proportion of nodules are left unclassifiable using ATA,^20,21,26,27^ which also uses a pattern-based approach, while Chng et al had a small number of cases non-classifiable with both BTA and ATA compared to TIRADS. A point-based system such as TIRADS is perhaps less likely to present this difficulty and all nodules were classifiable using TIRADS in the present cohort.

FNA is not without risk or discomfort and generates patient anxiety. Furthermore, it can often yield a non-diagnostic, Thy1, result or a Thy3 cytology outcome, *i.e*. “neoplasm possible” neither definitively benign or malignant – creating further uncertainty. BTA grading would have resulted in a higher rate of FNA in ultimately benign nodules than ACR-TIRADS or AI-TIRADS. ACR and AI-TI-RADS combine nodule diameter with ultrasound grading to direct FNA threshold. This reduces the risk and cost of subjecting patients with benign nodules or indolent cancers to biopsy and treatment while the follow-up protocol for smaller nodules graded TR3-5 for up to 5 years allows subsequent identification of significant malignancies. In a study comparing seven guidelines, although Korean TIRADS, ATA and the National Comprehensive Cancer Network were more sensitive, ACR had the lowest rate of unnecessary FNA at 25.3%.^21^ Xu et al^28^ and Grani et al^26^ also found ACR-TIRADS to perform best in this respect when comparing with Korean and EU-TIRADS and also ATA and American Association of Clinical Endocrinologists in the latter. Meanwhile, AI-TIRADS was more recently shown to avoid the highest number of unnecessary FNAs compared to Korean, European and ACR TIRADS.^22^ The phrase unnecessary FNA is, in reality, an over simplification as by nature it is established in retrospect. It functions as a comparative tool when assessing pros and cons of various classifications although does not entirely reflect the nuances of interpretation and decision making at the time of ultrasound evaluation. Moreover, measures to improve moderately high non-diagnostic cytology rates would enhance the overall efficacy of FNA. Regarding the follow-up schedule for select nodules with TIRADS systems, there are conceivable geographical, communication and compliance issues. In our experience, however, patients with non-diagnostic or indeterminate cytology or who are borderline surgical candidates often undergo ultrasound follow-up with no clear consenus on frequency or duration.

Although a fewer number of malignant nodules met criteria for FNA with the TIRADS systems compared to BTA, the majority met conditions for follow-up ultrasound, including a false-negative nodule graded BTA U2. A proportion of malignancies in our cohort would have required no FNA or follow-up with TIRADS. Hoang et al^29^ also found a small percentage of malignant nodules (2.5%) in this category when applying ACR-TIRADS in 100 consecutive nodules with definitive cytology or resection. Three false-negative nodules with TIRADS were mixed composition nodules with isoechoic solid components. This is a recognised pitfall,^8,30^ particularly for papillary cancers. In fact, two of these had a mural nodule forming acute angles with the cyst wall – a recognised suspicious finding according to ACR-TIRADS out with the standard reporting lexicon and should in fact be treated as suspicious.

The current study differs from the majority of the others in that we included a small number of nodules 5–10 mm in our cohort (7%). Besides TIRADS, other guidelines including ATA and European Society for Medical Oncology^31,32^ do not recommend FNA in nodules below 1 cm, risk factors and individual circumstances aside. However, these small nodules are still frequently encountered, often require classification in the ultrasound report and in the case of a TR5 nodule between 5 and 10 mm, *for example,* merit ultrasound follow-up. Two subcentimetre malignant nodules graded TR4 in our cohort were below FNA and follow-up threshold. While deemed moderately suspicious, such nodules at this size are highly likely to display indolent behaviour.

No ultrasound stratification tool has demonstrated perfect accuracy for predicting thyroid nodule malignancy. There is almost always a compromise between sensitivity and specificity and a degree of overlap between the appearances of benign and malignant nodules. An ideal system will allow the balance to be struck between identifying biologically relevant disease while avoiding over investigation of benign disease and overdiagnosis of subclinical cancers.

Study limitations include the relatively small number of included nodules, the retrospective nature and the fact that nodules were scored by two subspecialty radiologists with a moderately high level of experience in consensus. A prospective study with a larger patient cohort and an increased number of operators may be of more value where inherent difficulties in reviewing static ultrasound images would also be avoided. Using only cases with histology results meant that the observers were aware that the nodules being assessed had been subsequently resected in the vast majority of cases. This may have introduced bias towards the examined nodules being more likely to be malignant however hemithyroidectomy is a relatively common diagnostic procedure for a nodule with indeterminate ultrasound grading or cytology. Furthermore, this would have impacted on each of the grading systems equally and should not affect their comparison. No formal analysis was made of inter- or intraobserver variation. However, as the diagnostic performance was generally comparable between the three classifications it may come down to personal preference and perceived “user-friendliness” of a pattern-based or a point-based system. In real world medical practice, there may be clinical and practical reasons to have a lower threshold to proceed initially to FNA rather than recommend interim ultrasound follow-up for smaller nodules.

Regarding 218 thyroid nodules with histological correlation, BTA, ACR-TIRADS and AI-TIRADS classifications all had high sensitivity and NPV for malignancy. A proportion of nodules were difficult to classify using BTA. Either ACR-TIRADS or AI-TIRADS application would have resulted in a reduction of FNA in benign nodules.
